# Daily Life in a Dental Infirmary

**Published:** 1887-05

**Authors:** 


### Editorial, Etc.

---

DAILY LIFE IN A DENTAL INFIRMARY.—The reporter of one of the prominent daily papers gives his experience as a sight-seer in a Dental Infirmary, and as his account will answer as a description of the scenes of such an institution generally, we give the following extracts:

"The exterior of the building is so harmless and inoffensive in appearance that few persons would suspect that behind those walls are concealed something like one hundred and fifty human beings, the majority of them young men, engaged in mastering the technicalities connected with the preservation and extraction of molars, bicuspids, incisors, canines and all the other things in the mouth to which the scientists have given such terrible names, and which, on occasion, ache so like the late Samuel Hill. Is it not a dreadful thought? One hundred and fifty men about to be turned loose upon the inhabitants of this city, to establish 150 dental rooms, to smile blandly upon 150 patients who come timidly in, holding on to their jaws; to pop them into 150 chairs and say "Open your mouth—a little wider, please;" then silence for a moment; then 150 shrieks to rend the air, followed presently by an equal number of long-drawn "ahs!" expressive of immeasurable relief. It almost takes away one's breath to think of it.

But in the meantime, before those students become full-fledged dentists, they must have patients to practice on. And as few persons care to pay fancy prices for the sake of advancing the general cause of dental science, while at the same time incurring the risk of having their jaws broken or of inadvertently swallowing improved styles of forceps, it follows that the faculty of this institution has made the charge for all work done there very low (merely enough to cover the cost of the material used) to afford the necessary amount of inducement. Knowing, therefore, that visitors were not so infrequent as to excite comment, and observing several persons entering with that uncertain gait and troubled expression of countenance which marks the dentist's prey, a reporter, backed up by an intrepid artist, ventured into this place the other day to see what there was to be seen. The rumors afloat concerning the sad fate of various rash intruders into the stronghold of these embryo alveolar-tissue lacerators were powerless to deter them. Armed with an accident policy for one day, and with note-books and pencils carefully concealed, they ascended a flight of stairs and entered a long, narrow room containing about a dozen chairs of a kind but too familiar to need description. They were occupied by twelve unfortunates, mostly females, in various stages of unhappiness. There wasn't cheerfulness enough diffused over all their countenances put together to make up one single sickly defeated-candidate-the-day-after-election smile. But there was enough cheerfulness on the faces of the operators, as well as on those of the other students who were visible, to rather more than overbalance the gloom that otherwise characterized the proceedings. The reporter was very kindly received by an elderly gentleman with keen eyes and an encouraging smile, whose affable reception of visitors had a noticeably tranquillizing effect upon their nerves. Every facility was afforded for examining into the workings of the institution.

By rare good fortune the artist recognized a friend in a six foot student, who was watching a young man run a buzz-saw between the teeth of a neatly dressed and rather attractive young lady. He consented to act the part of cicerone, and under his guidance and protection the twain made the tour of the premises, beginning with the extracting-room. That is, they began with it after a while, but not quite immediately, for on trying the door it proved to be locked, and various hollow groans and muffled cries from beyond conveyed but too plainly to the listeners the fact that some student was adding to his information as to the ratio between his dexter arm and some decayed premaxillary grinder. When the student had concluded his operations the door was opened. About a dozen students were gathering around the operating-chair, and a young woman in a state of collapse was departing. The students seemed to be having a little discussion as to whose turn it was to have the next "crack" and waved various dreadful-looking instruments of torture about them in an alarmingly careless manner.

Leaving this ensanguined field the exploring party ventured next into the third story. Here was another large room, with more chairs and more unfortunates. All the chairs were full and the students were working away for dear life. The professors look at the patient's teeth before the student begins to operate and after he has completed his work. If it is satisfactory he receives a mark in accordance with the excellence of his work. If it is not good he has to take it out and do it over again. This, the reporter was informed, did not very often happen, but when it did it was apt to engender ill-feeling between the operator and operatee. Adjoining this was a large room devoted to the manufacture of plates and sets of teeth. In one corner of the room was a furnace about as hot as a crematory to full blast. The student led the unhappy visitors directly to this, and planted them within six inches of it while he gave them all the details he could think of concerning the institution.

The Professors afterwards took the visitors in charge and showed them the museum, containing among other things casts of the heads of persons upon whom remarkable dental and surgical operations had been performed by the faculty of this institute. There was the case, for instance, of a young man whom they called "Tommy," who, through some cancerous affection, had lost his entire nose and all his teeth. As shown by the cast, he must have been a hideous object to gaze upon. The middle finger of his left arm was bound down upon his face, the skin opened and left there until it should unite. When union had taken place the finger was cut off at the first joint, and the man had a very tolerable-looking nose at the expense of a finger.

The Professor was interrogated as to the nationality of the students. He replied that they came from all parts of the world. The course takes two years. Each student works three days filling teeth, two days making plates, and one day at extracting. This, with the lectures and subsequent copying of his notes and reading text-books, fills out his week very thoroughly. The course embraces, besides instruction in dentistry, a complete course of anatomy and the science of medicine. One would naturally suppose that the institute would be patronized almost exclusively by the poorer classes, but this, the Professor stated, was not the case. Many persons who were abundantly able to pay the highest prices came here for the reason, he presumed, that they were sure of getting honest work. Another class of visitors whom they would like to exclude, but could not, were young women of flashy attire and not irreproachable morals. Most of them had nothing the matter with their teeth, and just wanted them cleaned a little to give them a chance to make the acquaintance of the "boys."

All the students carry about with them little diagrams which look like a small stockade-fort erected for protection against the Indians. But it is intended to represent all the teeth in the human head, first and second sets. Every patient receives one of these pictures, with the tooth or teeth to be operated on marked on it by the Professor, and the student works with this as a guide. He cannot touch a tooth that is not marked on this chart.

While the reporter was examining a chart that had 17 teeth marked to come out, a student with a very determined expression of countenance took the card from him, under the impression that he had just received it in the regular way from a professor, and, with a smile intended to be reassuring, sought to crowd him down into an operating chair. The reporter fled with never a backward glance.

